# A lupus anti-DNA autoantibody mediates autocatalytic, targeted delivery of nanoparticles to tumors

**DOI:** 10.18632/oncotarget.11015

**Published:** 2016-08-02

**Authors:** Zeming Chen, Jaymin M. Patel, Philip W. Noble, Cesar Garcia, Zhangyong Hong, James E. Hansen, Jiangbing Zhou

**Affiliations:** ^1^ Department of Neurosurgery, Yale University, New Haven, CT 06510, USA; ^2^ State Key Laboratory of Medicinal Chemical Biology, College of Life Sciences, Nankai University, Tianjin 300071, P. R. China; ^3^ Department of Medical Oncology, Yale University, New Haven, CT 06510, USA; ^4^ Department of Therapeutic Radiology, Yale University, New Haven, CT 06510, USA; ^5^ Yale Cancer Center, Yale University, New Haven, CT 06510, USA; ^6^ Department of Biomedical Engineering, Yale University, New Haven, CT 06511, USA

**Keywords:** nanoparticles, autocatalysis, targeted delivery, anti-DNA autoantibody, breast cancer

## Abstract

Strategies to target nanoparticles to tumors that rely on surface modification with ligands that bind molecules overexpressed on cancer cells or the tumor neovasculature suffer from a major limitation: with delivery of toxic agents the amount of molecules available for targeting decreases with time; consequently, the efficiency of nanoparticle delivery is reduced. To overcome this limitation, here we propose an autocatalytic tumor-targeting mechanism based on targeting extracellular DNA (exDNA). exDNA is enriched in the tumor microenviroment and increases with treatment with cytotoxic agents, such as doxorubicin (DOX), due to release of DNA by dying tumor cells. We tested this approach using poly(lactic-co-glycolic acid) (PLGA) nanoparticles surface-conjugated with fragments of 3E10 (3E10^EN^), a lupus anti-DNA autoantibody. We demonstrated that 3E10^EN^-conjugated nanoparticles bound to DNA and preferentially localized to tumors *in vivo*. The efficiency of tumor localization of 3E10^EN^-conjugated, DOX-loaded nanoparticles increased with time and subsequent treatments, demonstrating an autocatalytic effect. 3E10^EN^-conjugated DOX-loaded nanoparticles exhibited a significant anti-tumor effect that was superior to all controls. This work demonstrates the promise of autocatalytic drug delivery mechanisms and establishes proof of concept for a new anti-DNA autoantibody-based approach for enhancing delivery of nanoparticles to tumors.

## INTRODUCTION

The first nanodrug, Doxil®, which is a formulation of doxorubicin (DOX) in liposomal nanocarriers, was approved by the FDA for treatment of human patients with AIDS-related Kaposi's sarcoma in 1995 [1]. Since then, development of nanocarriers for delivery of chemotherapeutic drugs has emergered as a promising approach to cancer therapy with many advantages compared to free drugs. Due to the enhanced permeability and retention (EPR) effect resulting from the size difference between interendothelial junctions in tumors (40-80 nm) and healthy tissue (<8 nm) and defective lymphatic drainage in tumors [2], the use of nanocarriers alters the bio-distribution of the encapsulated drugs and results in preferential accumulation in tumors. However, this passive targeting approach based on the EPR effect may not be sufficient to yield meaningful gains in cancer therapy [3]. To further enhance targeting efficiency, nanocarriers have been engineered through conjugation to ligands that have high affinities for molecules overexpressed in cancer cells or the tumor neovasculature or tumor microenvironment. [4–8]. Nonetheless, these traditional targeting approaches suffer from a significant limitation: with the delivery of chemotherapeutic agents which kill the tumor cells and neovasculature, the amount of molecules avaliable for targeted delivery decreases with time; consequently, the efficiency of nanoparticle accumulation in tumors is reduced.

A key feature that distinguishes the microenvironment within tumors from that of healthy tissue is the presence of a comparatively larger amount of extracellular DNA (exDNA) [9–12], which originates from actively dividing, apoptotic or necrotic tumor cells and neutrophil extracellular traps [12–14]. Importantly, the amount of exDNA in the region of tumors further increases during treatment with cytotoxic agents, such as DOX, that cause tumor cell death and release of DNA [15, 16]. The greater concentration of exDNA in the tumor environment compared to normal tissues offers an opportunity to develop a novel tumor targeting approach using an agent that has a high affinity with DNA. We believe that an anti-DNA autoantibody associated with the autoimmune disease systemic lupus erythematosus (SLE) is well suited to this task.

Circulating autoantibodies that bind DNA are commonly found in patients with SLE. The role of these anti-DNA autoantibodies in SLE pathophysiology is unclear, but we have recently recognized the potential to harness them for use in cancer therapy [16–19]. Most important for the present work is the finding that a specific nuclear-penetrating lupus anti-DNA autoantibody, 3E10, has great potential to be used as a tumor-targeting agent for nanocarriers. 3E10 penetrates cell nuclei and inhibits DNA repair in a manner that is not toxic to normal cells but can kill cancer cells with defects in DNA repair [16]. The ability of 3E10 to penetrate nuclei is dependent on the presence of exDNA, and when administered to mice and rats 3E10 is preferentially attracted to tissues in which exDNA is enriched, including tumors, regions of ischemic brain in stroke models, and skeletal muscle subject to contractile injury [9, 20–22].

**Figure 1:**
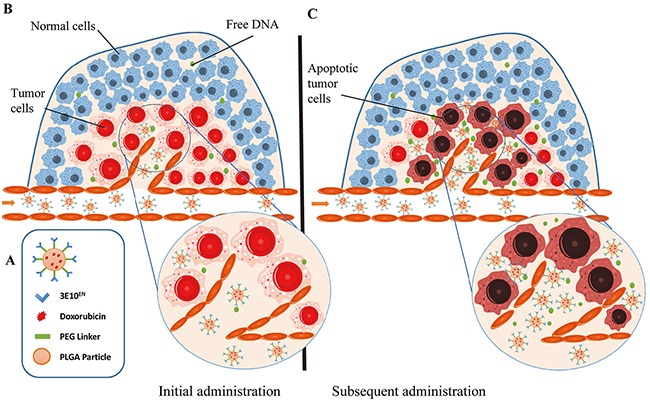
Schematic of autocatalytic, tumor-targeted delivery of nanoparticles by 3E10^EN^ **A.** Schematics of PLGA nanoparticles with surface conjugation of 3E10^EN^ for exDNA targeting and with internally encapsulated DOX. **B, C.** Mechanism of autocatalytic, tumor-targeted delivery of nanoparticles. 3E10^EN^ has the ability to home nanoparticles to tumors, which contain a greater amount of exDNA than healthy tissue (B). The concentration of exDNA in tumor environments is expected to further increase with time and delivery of cytotoxic therapy, such as DOX. Therefore, the efficiency of nanoparticle accumulation in tumors autocatalytically increases with time and subsequent treatments (C).

Based on the capacity of 3E10 to home to sites of exDNA, we proposed to use this antibody to develop an autocatalytic, tumor-targeting mechanism for systemic delivery of nanoparticles to tumors by targeting exDNA with surface-conjugated fragments of 3E10. Because the concentration of exDNA in tumor environments is expected to increase with time and delivery of cytotoxic therapy, by using this strategy it is expected that the efficiency of nanoparticle accumulation in tumors will autocatalytically increase with time and subsequent treatments (Figure 1). To test this approach, in the present study we generated poly(lactic-*co*-glycolic acid) (PLGA) nanoparticles with 3E10 fragments conjugated to the surface for exDNA targeting and with encapsulated DOX as a model chemotherapy drug. We show that the resulting nanoparticles associate with exDNA, preferentially localize to tumors in an autocatalytic manner, and yield significant suppression of tumor growth in a syngeneic mouse breast cancer model.

## RESULTS

### Synthesis and characterization of nanoparticles with and without surface anti-DNA autoantibody

In order to avoid potential nonspecific toxicity secondary to Fc-mediated activation of complement or antibody-dependent cell-mediated cytotoxicity, 3E10 fragments lacking an Fc region have recently been generated and tested. One of the most promising variants of these fragments is an enhanced di-single chain variable fragment of 3E10 that has been mutated to improve its binding affinity for DNA [17]. We therefore chose this fragment, 3E10 (D31N) di-scFv, for testing in the present study and it is hereafter referred to as 3E10^EN^ (EN=enhanced).

**Figure 2:**
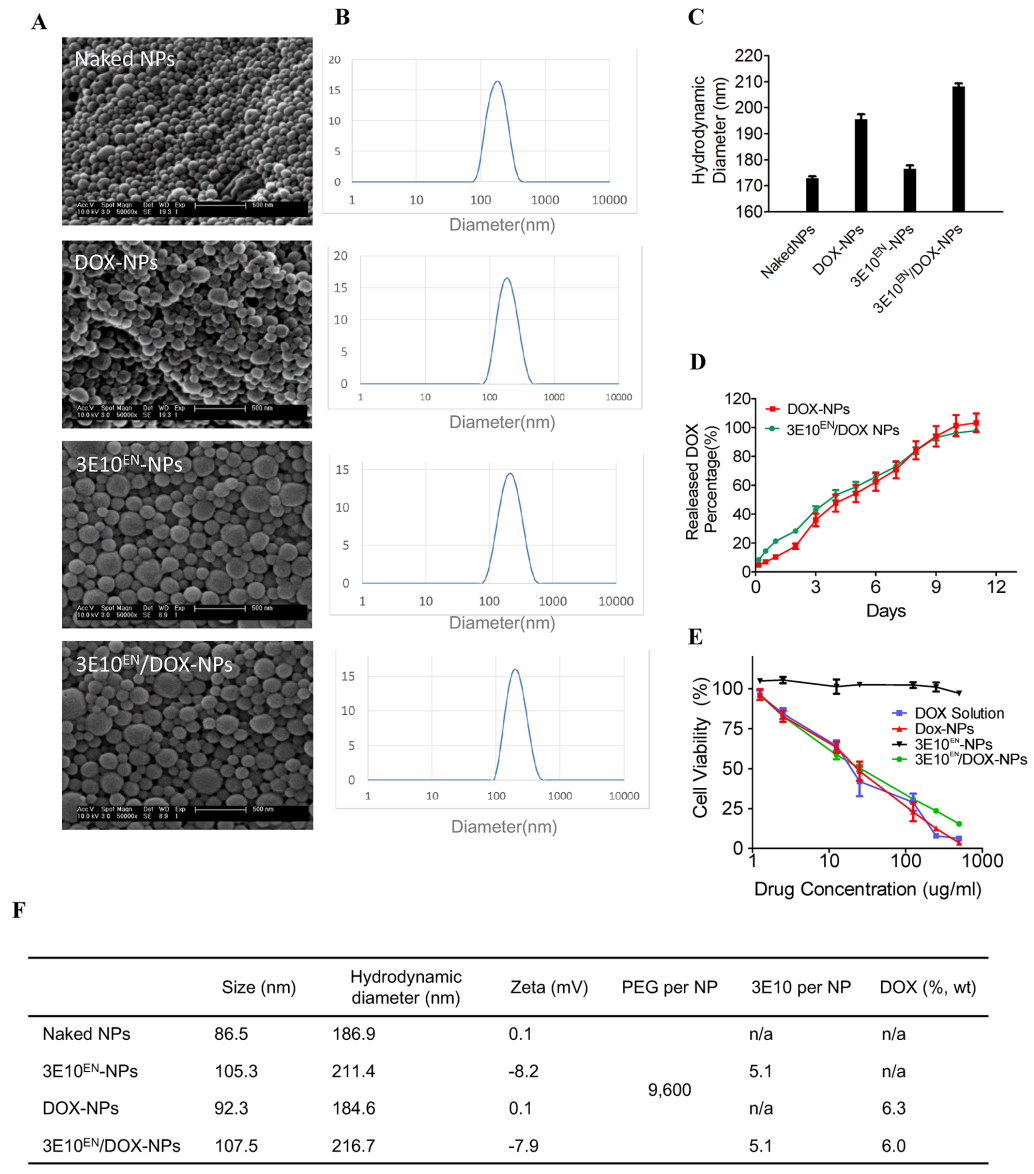
Characteristics of nanoparticles **A.** Morphology of nanoparticles used in this study as captured by SEM. **B, C.** Hydrodynamic diameters of nanoparticles as determined by DLS. **D.** Controlled DOX release profiles of DOX-NPs and 3E10^EN^/DOX-NPs. **E.** Effects of nanoparticles on the viability of 4T1 murine breast cancer cells. The cells were treated for three days with DOX alone, DOX-NPs, 3E10^EN^-NPs, or 3E10^EN^/DOX-NPs and then survival was evaluated by MTT assay. Percent cell viability is shown. **F.** Summary of the major characteristics of the nanoparticles used in this study.

To test the ability of 3E10^EN^ to autocatalytically deliver nanoparticles to tumors we synthesized DOX-loaded PLGA nanoparticles with surface-conjugated 3E10^EN^ (3E10^EN^/DOX-NPs) as shown in the schematic in Figure 1A. PLGA was first conjugated with poly(L-lysine) (PLL) and the resulting PLGA-PLL, which contains lysine groups for surface functionalization, was used as the starting material. To enable efficient encapsulation, the hydrochloride group in commercial DOX hydrochloride was removed through titration using triethylamine in dichloromethane. Nanoparticles were synthesized through the standard single emulsion procedure and further modified with NHS-PEG-Mal to display maleimide groups for conjugation of thiolated 3E10^EN^. Controls included nanoparticles without surface conjugated 3E10^EN^ (DOX-NPs), nanoparticles with 3E10^EN^ but without DOX (3E10^EN^-NPs), and nanoparticles without 3E10^EN^ or DOX (naked NPs) and were synthesized using the same procedures but without 3E10^EN^ conjugation, DOX encapsulation, or both. Schematic diagrams of PLGA-PLL synthesis and NP fabrication are shown in Supplementary Figure S1. Scanning electron microscopy (SEM) showed that all nanoparticles were spherical and of size 86-107 nm (Figure 2A, 2F). The hydrodynamic diameters of the nanoparticles of different formulations were in the range of 180-210 nm (Figure 2B, 2C). The conjugation of 3E10^EN^ slightly increased nanoparticle size. An average of 5 3E10^EN^ molecules were conjugated to the surface of each nanoparticle. DOX was encapsulated with 6.0% by weight (Figure 2F). Zeta potentials of the different NPs were also measured. PLGA NPs with PLL were found to have a neutral surface charge, whereas conjugation of 3E10^EN^ decreased the surface charge to −8 mV (Figure 2F).

We compared the release of DOX from 3E10^EN^/DOX-NPs and DOX-NPs to determine if 3E10^EN^ interfered in any way with drug release. As shown in Figure 2D, DOX was released in an equivalent controlled manner over 12 days from both 3E10^EN^/DOX-NPs and DOX-NPs, indicating that 3E10^EN^ did not interfere with drug release. Next, the nanoparticles were tested for effects on the viability of 4T1 murine breast cancer cells. The cells were treated for three days with DOX alone, DOX-NPs, 3E10^EN^-NPs, or 3E10^EN^/DOX-NPs and then survival was evaluated by MTT assay. Free DOX, DOX-NPs, and 3E10^EN^/DOX-NPs all yielded comparable inhibition of cells, while the 3E10^EN^-NPs without DOX were not significantly toxic to the cells (Figure 2E). The characteristics of the nanoparticles used in this study are summarized in Figure 2F. These results are of particular interest because, although the amount of DOX released from the NPs during the treatment period was approximately 40% of the amount of drug to which cells were exposed in the group that received treatment with free DOX, the DOX-NPs and free DOX had a similar effect on cell viability. This may seem surprising at first, but similar findings have been extensively documented in the literature [23–27] and this effect is likely due to differences between the mechanisms governing cellular uptake and export of nanoparticles and free drugs. For example, free DOX, but not DOX-loaded nanoparticles, is a substrate of ATP-binding cassette (ABC) transporters highly expressed in tumor cells.

### 3E10^EN^-conjugation enhances the interaction of nanoparticles with exDNA

**Figure 3:**
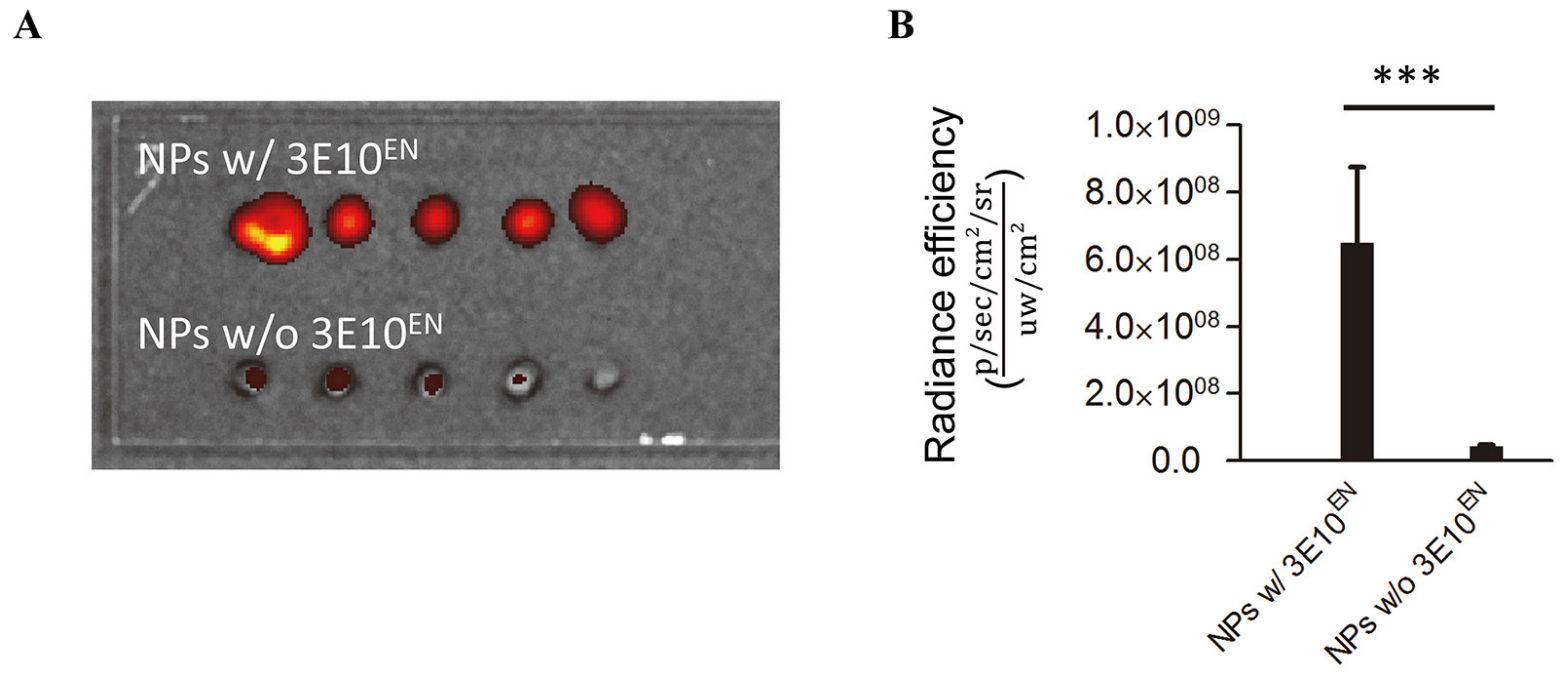
3E10^EN^ conjugation enhances the interaction of nanoparticles with DNA A glass slide coated with linearized plasmid DNA was incubated with nanoparticles with or without surface 3E10^EN^. For the purpose of detection the nanoparticles were encapsulated with IR780. After a 60-minute incubation with the nanoparticles the slide was rinsed and the signal of IR780, which correlated with the amount of nanoparticles, was visualized by IVIS. Representative images are shown in **A.** and quantitative analysis of nanoparticles binding to the slide based on fluorescence intensity is shown in **B.** ***: *P* < 0.001.

We previously demonstrated that 3E10 has a high affinity with DNA [9, 16], and we therefore hypothesized that surface-conjugated 3E10^EN^ would enhance the interaction of nanoparticles with DNA. To test this we coated a glass slide with linearized plasmid DNA and incubated it with nanoparticles with and without surface 3E10^EN^. For the purpose of detection, the nanoparticles were encapsulated with IR780, a near-infrared fluorescent dye. After a 60-minute incubation with the nanoparticles the slide was rinsed and the signal of IR780, which correlated with the amount of nanoparticles, was visualized by IVIS. Representative images are shown in Figure 3A, and signal was quantified in Figure 3B. Conjugation of 3E10^EN^ increased the association of nanoparticles with the DNA-coated glass surface by 5.6 fold, confirming that 3E10^EN^-NPs are attracted to DNA as expected.

### exDNA is enriched in 4T1 tumors and increases with 3E10^EN^/DOX-NP treatment

**Figure 4:**
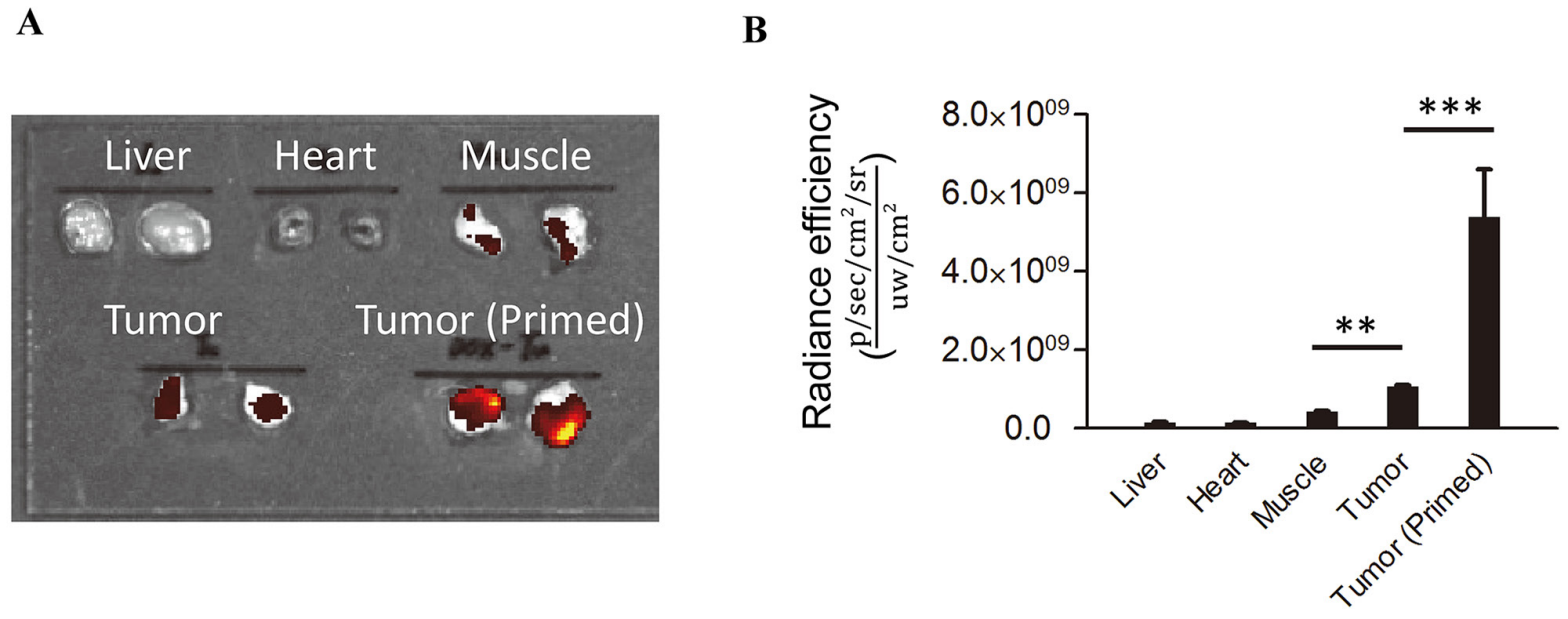
exDNA is enriched in tumors The relative amounts of exDNA in normal tissues and 4T1 tumors with and without treatment with 3E10^EN^/DOX-NPs was determined by Picogreen staining. The amount of exDNA in untreated tumors was 7.5, 11.7 and 2.5 times greater than what is found in the liver, heart, and muscle. Treatment of the mice with 3E10^EN^/DOX-NPs increased the amount of exDNA in tumors by 5.1 fold compared to tumors in untreated mice. A representative image of exDNA in indicated tissues is shown in **A.** and a quantitative analysis of exDNA present in the indicated tissues (n=5) is shown in **B.** The data are presented as mean +/− SD. **: *P* < 0.01. ***: *P* < 0.001.

We next set out to test the ability of 3E10^EN^ to target nanoparticles to tumors using the syngeneic 4T1 murine breast cancer mouse model. In this model 4T1 breast cancer xenografts were generated by subcutaneous injection in BALB/c mice. Prior to initiating efficacy studies we first evaluated the relative amounts of exDNA in normal tissues and 4T1 tumors with and without treatment with 3E10^EN^/DOX-NPs by Picogreen staining. Consistent with previous findings [9–12], the amount of exDNA in untreated tumors was 7.5, 11.7 and 2.5 times greater than what is found in the liver, heart, and muscle (Figure 4A, 4B), suggesting exDNA is a viable target for preferential drug delivery to tumors in this model. We next tested the effect of treatment with 3E10^EN^/DOX-NPs on the amount of exDNA. Mice bearing 4T1 tumors were treated with intravenous injection of 3E10^EN^/DOX-NPs on two consecutive days, and then on the third day the mice were sacrificed and tumor exDNA content was evaluated. As shown in Figure 4A, 4B, treatment of the mice with 3E10^EN^/DOX-NPs increased the amount of exDNA in tumors by 5.1 fold compared to tumors in untreated mice. Taken together, our results confirmed that this mouse model was appropriate for testing the proposed autocatalytic delivery of nanoparticles to tumors using 3E10^EN^ to target exDNA.

### 3E10^EN^ mediates autocatalytic, tumor-targeted delivery of nanoparticles

**Figure 5:**
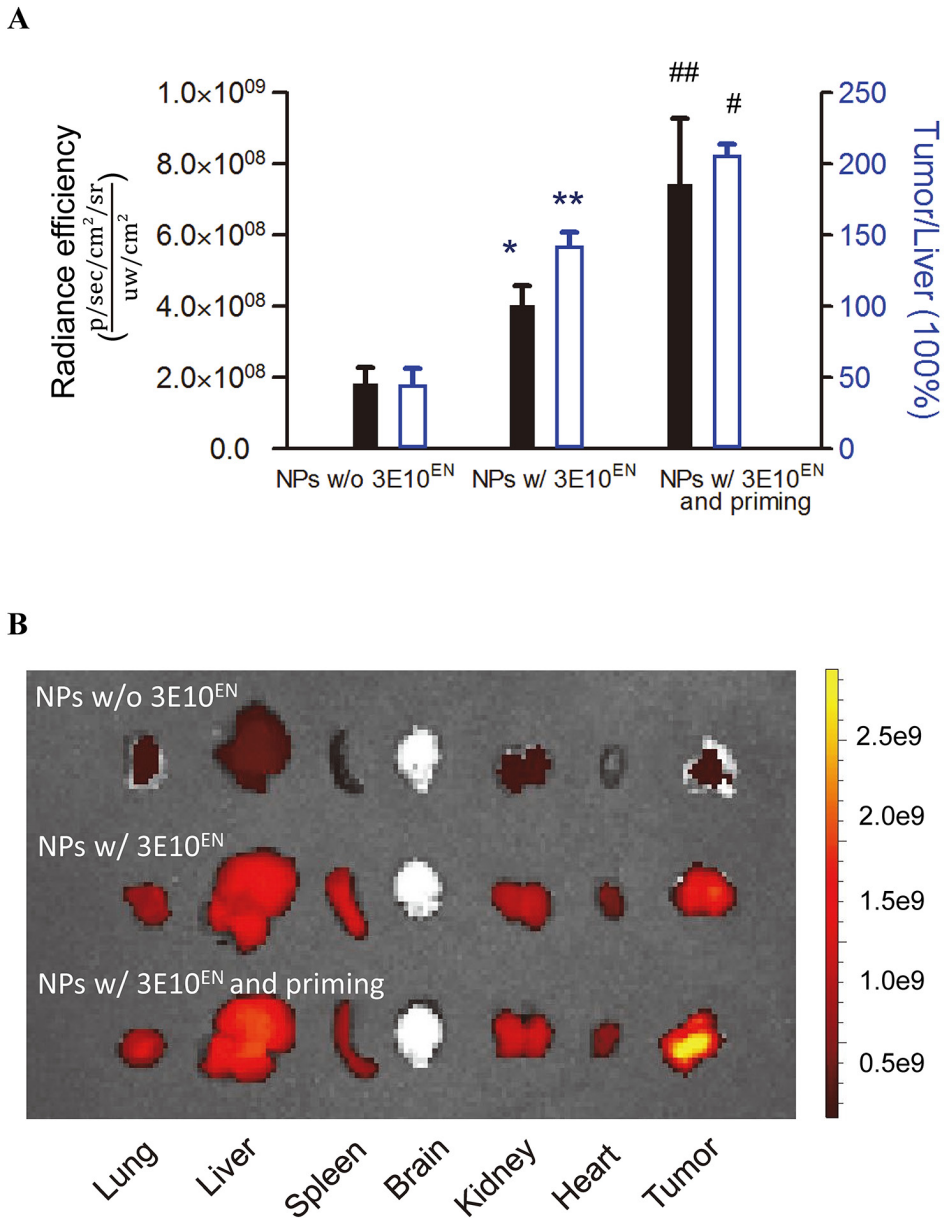
3E10^EN^ mediates autocatalytic, tumor-targeted delivery of nanoparticles IR780-loaded nanoparticles with or without 3E10^EN^ conjugation were administered intravenously to 4T1 tumor-bearing mice. Twenty-four hours later, tumors were excised and imaged using an IVIS imaging system. Naked NPs were observed to localize into a range of tissues. By contrast, 3E10^EN^-conjugated NPs showed a pattern of preferential uptake into tumors and a 2.3 fold increase in tumor localization compared to naked NPs. In addition, priming treatments with 3E10^EN^/DOX-NPs significantly enhanced tumor delivery of the nanoparticles. The average amount of nanoparticles in tumors from mice that received priming treatments was 1.8 times greater than the amount in tumors from mice without priming. With priming, the accumulation of nanoparticles in tumors was 4.1 times higher than that in the liver, compared to 0.5 times for mice that received treatment with naked NPs. Quantitative analysis of the accumulation of indicated nanoparticles in tumors (n=4) is shown in **A.** * and ^#^ represent statistical analyses between the NPs w/ 3E10^EN^ group and the NPs w/o 3E10^EN^ group, and between the NPs w/ 3E10^EN^ and priming group and the NPs w/ 3E10^EN^ group, respectively. * and ^#^: *P* < 0.05, ** and ^##^: *P* < 0.01. Representative IVIS images of the bio-distribution of nanoparticles are shown in **B.**

We next proceeded to determine if 3E10^EN^ can mediate preferential delivery of nanoparticles to tumors. IR780-loaded nanoparticles with or without 3E10^EN^ conjugation were administered intravenously to 4T1 tumor-bearing mice. Twenty-four hours later, tumors were excised and imaged using an IVIS imaging system. Naked NPs were observed to localize into a range of tissues, with some tumor uptake but the greatest amount of uptake was seen in the liver. By contrast, 3E10^EN^-conjugated NPs showed a pattern of preferential uptake into tumors rather than liver and a 2.3-fold increase in tumor localization compared to naked NPs (Figure 5A). 3E10^EN^ did not alter the circulation life of NPs (Supplementary Figure S2), and therefore the enhanced uptake mediated by 3E10^EN^ is most likely due to interaction with exDNA in tumors. These results suggest preferential targeting of nanoparticles to untreated tumors by 3E10^EN^.

According to our proposed strategy, the efficiency of 3E10^EN^-mediated delivery should increase autocatalytically with time and delivery of treatments that induce release of tumor DNA and cause further accumulation of exDNA in the tumor environment (Figure 1). To test this hypothesis, we treated 4T1 tumor-bearing mice with 3E10^EN^/DOX-NPs without IR780 daily for two consecutive days (referred to here as the priming treatments). On the third day, mice received a final injection of IR780-loaded 3E10^EN^-NPs. Twenty-four hours later, tumors were excised and imaged. As shown in Figure 5A, the priming treatments with 3E10^EN^/DOX-NPs significantly enhanced the tumor delivery of the nanoparticles. The average amount of nanoparticles in tumors from mice that received priming treatments was 1.8 times greater than the amount in tumors from mice without priming. Notably, with priming, the accumulation of nanoparticles in tumors was 4.1 times greater than that in the liver, compared to 0.5 times for mice that received treatment with naked NPs.

### 3E10^EN^/DOX-NPs have a significantly greater effect on tumors than DOX-NPs or DOX alone

**Figure 6:**
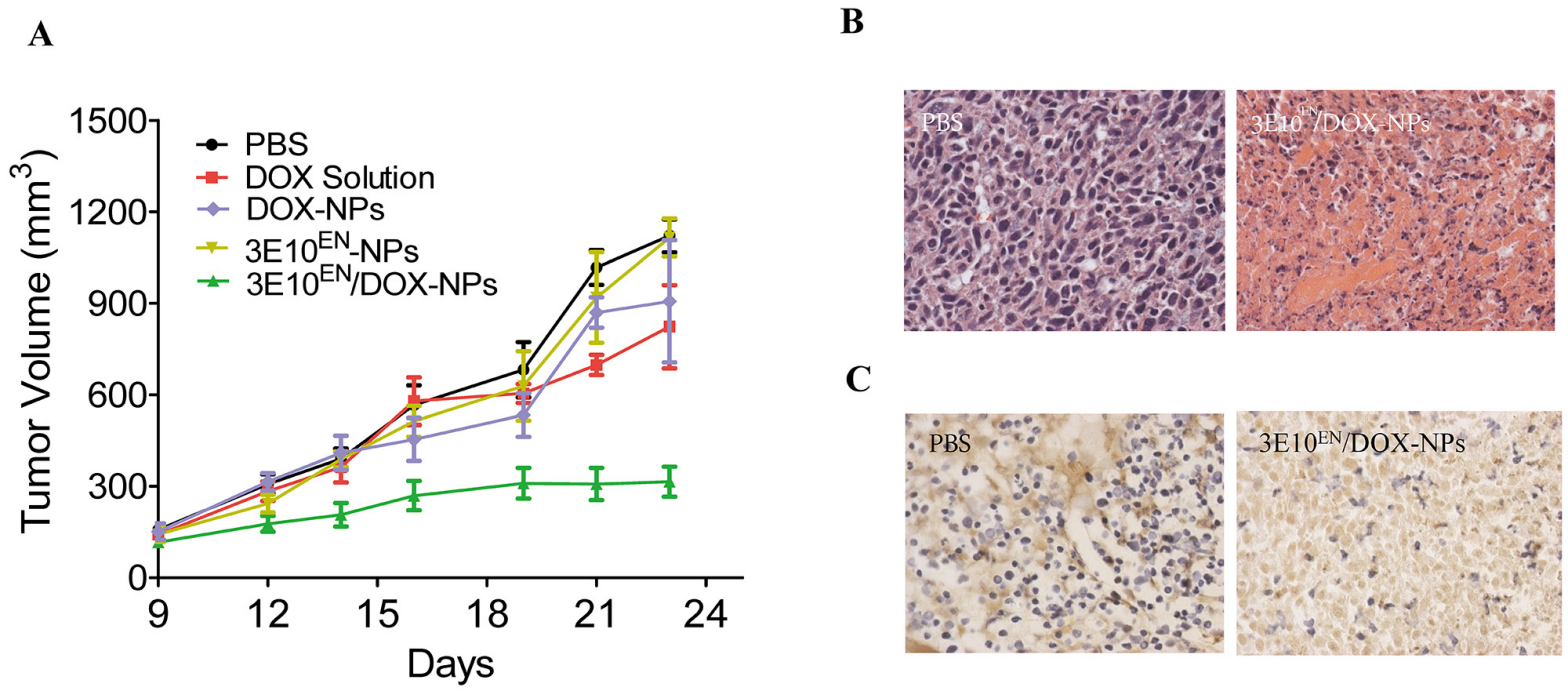
3E10^EN^/DOX-NPs have a significant effect on tumors **A.** Effect of nanoparticles on 4T1 tumor growth. Mice bearing 4T1 tumors of ~100 mm^3^ size were treated three times a week with intravenous injection of control PBS, free DOX, DOX-NPs, 3E10^EN^-NPs, or 3E10^EN^/DOX-NPs (n=7 mice per group). Tumor volumes were measured three times a week, and the resulting growth curves are shown. **B.** H&E staining of tumors with and without 3E10^EN^/DOX-NP treatment. Tumors from control treatments revealed a highly cellular mass with prominent nuclei; in contrast, tumors from animals treated with 3E10^EN^/DOX-NPs exhibited a much lower cellular mass and a lower nuclear-cytoplasmic ratio. **C.** TUNEL staining of tumors with and without nanoparticle treatment. Tumors in mice treated with 3E10^EN^/DOX-NPs were found to have a marked increase in the number of apoptotic cells.

Lastly, we assessed whether the 3E10^EN^-based approach to targeting exDNA for autocatalytic tumor-targeted drug delivery would result in improvements in tumor response to treatment *in vivo*. Mice bearing 4T1 tumors of ~100 mm^3^ size were treated three times a week with intravenous injection of control PBS, free DOX, DOX-NPs, 3E10^EN^-NPs, or 3E10^EN^/DOX-NPs. Tumor volumes were measured three times a week, and the resulting growth curves are shown in Figure 6A. Of all of the treatment groups, only the 3E10^EN^/DOX-NPs were observed to greatly inhibit tumor growth (p<0.01 compared to both free DOX and DOX-NPs). By the end of the study, compared to the control group that received PBS treatment, treatments with free DOX or DOX-NPs reduced tumor volumes by only 26% and 19%, respectively. No significant difference was found between these two groups. By contrast, treatment with 3E10^EN^/DOX-NPs reduced tumor volume by 72%. Histologically, tumors from control treatments revealed a highly cellular mass with prominent nuclei; in contrast, tumors from animals treated with 3E10^EN^/DOX-NPs exhibited a much lower cellular mass, a lower nuclear-cytoplasmic ratio (Figure 6B), and a marked increase in the number of apoptotic cells measured by TUNEL staining (Figure 6C).

## DISCUSSION

In this study we proposed a novel autocatalytic tumor-targeting mechanism for systemic delivery of nanoparticles to tumors that takes advantage of exDNA in the tumor environment. We tested this approach using 3E10^EN^ as the targeting ligand for exDNA and DOX as the model drug, and have now shown that 3E10^EN^ mediates efficient delivery of nanoparticles to tumors and that this efficiency increases with subsequent treatments as more exDNA is released by the dying tumors. Compared to other targeting approaches that suffer from reduced efficiency over time as the relevant targets regress with treatment, the present technique has the key advantage of improved efficiency with time and treatment due to increased release of exDNA into the target environment. Therefore, the proposed mechanism may represent a novel direction for the development of nanocarriers for targeted drug delivery.

The present work also reveals a new application for the 3E10 antibody in molecular therapy. 3E10 has the unusual ability to penetrate into the nuclei of living cells, and the antibody has previously been used to deliver therapeutic cargo proteins *in vitro* and *in vivo* [28, 29]. More recently, the ability of 3E10 to inhibit DNA repair has been recognized as having potential for use against DNA repair-deficient tumors [16, 17, 19]. Our present work took advantage of the capacity of 3E10 to home to sites of exDNA *in vivo* by using it to guide the nanoparticles to tumor sites in an autocatalytic manner. This work establishes proof of concept for an anti-DNA autoantibody-based approach to targeting cargo molecules including nanoparticles to sites of exDNA, which is relevant to the treatment of cancer as well as ischemic or traumatic conditions such as stroke, infarction, or injury wherein DNA is released at the site of damage.

In conclusion, nanomedicine has the potential to make major contributions to clinical cancer care. In the meanwhile, select lupus anti-DNA autoantibodies have emerged as possible new agents for use in cancer therapy due in part to their affinity for DNA. Based on our present work, we believe strategies to combine DNA-targeting antibodies with nanocarriers will help facilitate translation of both technologies into clinically relevant therapies.

## MATERIALS AND METHODS

### Materials and cell culture

All chemicals were purchased from Sigma-Aldrich unless otherwise noted. The mouse mammary tumor cell line 4T1 was obtained from American Type Culture Collection (ATCC) and cultured in DMEM medium (Invitrogen) supplemented with 10% fetal bovine serum (Invitrogen), 100 units/ml penicillin and 100 ug/ml streptomycin (Invitrogen), in a 37°C incubator containing 5% CO2.

### Synthesis of PLGA-PLL

PLGA-PLL was synthesized according to procedures that we previously reported [6, 30]. Briefly, PLGA (3 g, 50:50 PLGA Acid End Group; i.v. ~0.67 dL/g; Absorbable Polymers, AL) and 200 mg poly(ε-carbobenzoxyl-L-lysine) (PLL) (MW 1000-4000 Da, Sigma) were dissolved in 6 mL dimethlyformamide in a dry round-bottom flask under argon. Dicyclohexyl carbodiimide (58 mg) and 0.31 mg dimethylaminopyridine in 2 mL dimethlyformamide was added to the polymer solution and allowed to stir for 48 h. The reacted solution was diluted by the addition of chloroform and precipitated in methanol. The dried polymer was then re-dissolved in chloroform, precipitated in ether, and dried under vacuum for 24 h. To remove protection, dried protected product was dissolved in 10 mL hydrogen bromide, 30% wt in acetic acid and allowed to stir for 90 min for deprotection. The polymer was precipitated in ether and washed until the product changed from a yellow to an off-white appearance. The product was then dissolved in chloroform and precipitated in ether. The polymer was vacuum dried for 24 h to remove all traces of ether. Samples before and after deprotection were collected to confirm modification of the polymer and subsequent removal of protecting carbobenzoxyl groups. The samples were dissolved in trifluoroethanol and evaluated from 200 to 350 nm using spectroscopy (Cary 50 Bio UV-Vis Spectrophotometer, Varian, Palo Alto, CA).

### 3E10^EN^ production and thiolation

3E10 (D31N) di-scFv (referred to as 3E10^EN^ in this study) was produced in and purified from *P. pastoris* as previously described [17]. Purity and identity of the 3E10^EN^ isolated from *P. pastoris* supernatant was confirmed by SDS-PAGE and anti-Myc Western blot prior to conjugation to nanoparticles.

Thiolation of 3E10^EN^ antibody was performed using Traut's agent. Briefly, 54 uL 3E10 solution (5 mg/ml) and 18 uL Traut's agent solution (10 mg/ml) were added to 1 ml PBS (pH 8.0 with 5 mM EDTA). The thiolation process took 1 hour by rotating the mixed solution on a horizontal shaker at room temperature.

### Nanoparticles synthesis

To produce DOX for nanoparticle synthesis, the hydrochloride groups in commercial DOX hydrochloride (Sigma) were removed through titration using triethylamine in dichloromethane, resulting in DOX soluble in organic solvents. DOX-loaded nanoparticles were synthesized according to the standard single emulsion procedure. For synthesis of DOX-NPs, 50 mg PLGA-PLL and 6.7 mg doxorubicin were dissolved in 2 mL ethyl acetate. The solution was then added dropwise to a solution of 2 ml 2.5% polyvinyl alcohol (PVA). The resulting emulsion was sonicated on ice 3 times for 10 seconds each. The emulsion was then poured into a beaker containing aqueous 0.3% (v/v) PVA and stirred at room temperature overnight to allow the EA to evaporate and the particles to harden. Particles were collected by centrifugation at 18000 rpm for 30 minutes, washed twice with water, frozen, and lyophilized. For synthesis of 3E10^EN^/DOX-NPs, the same emulsion procedures were used. After overnight evaporation, nanoparticles were collected and re-suspended in PBS containing NHS-PEG5000-Mal (8mg, JenKem Technology). After a 30 minute reaction, extra NHS-PEG5000-Mal was removed by centrifuge (18000 rpm, 30 min). PEGylated nanoparticles were then re-suspended in PBS containing thiolated 3E10 (270 ug). Sixty minutes later, nanoparticles were collected by centrifugation at 18000 rpm, 30 min, washed twice with water, frozen, and lyophilized. Naked NPs and 3E10^EN^-NPs were synthesized according to the same procedures but without DOX/3E10^EN^ and DOX, respectively.

### Characterization of nanoparticle size, morphology and Zeta potential

The morphology and size of nanoparticles was characterized by Scanning electron microscopy (SEM). Briefly, dry nanoparticles were mounted on carbon tape and sputter-coated with gold in an argon atmosphere using a sputter current of 40 mA (Dynavac Mini Coater, Dynavac, USA). SEM analysis was carried out with a Philips XL30 SEM using a LaB electron gun with an accelerating voltage of 3 kV.

The hydrodynamic diameter of nanoparticles was measured using Dynamic Light Scattering (DLS). A transparent cuvette was filled with 0.25 mg mL-1 nanoparticles in HPLC-grade water. The capped cuvette was placed in a Zetasizer (Malvern) and dynamic light scattering data was read. Zeta potential was also measured using the Zetasizer.

### Characterization of conjugation efficiency

Ten mg 3E10^EN^ nanoparticles was dissolved in 100 uL DMSO and added to 900 uL ddH2O to make a final concentration 10 mg/ml. The amount of 3E10^EN^ in the solution was determined by the standard BCA assay (Thermo Scientific). Nanoparticles without 3E10^EN^ processed using the same procedures were used as a control.

### Characterization of drug encapsulation

To determine the drug encapsulation efficiency (EE) and loading efficiency (LE), 1 mg of DOX-loaded nanoparticles were dissolved in 100 uL DMSO and added to 900 uL ddH2O to make a final concentration of 1 mg/ml. Nanoparticle solution was then spun briefly at 13,000 rpm and 100 uL supernatant was transferred to a microplate (BioTek). The fluorescence intensity was measured at 470 nm/590 nm (Ex/Em) and the concentration of DOX was calculated according to a standard curve of free DOX. EE and LE was calculated as follows:

$$\begin{array}{l} \text{Encapsulation Efficiency(EE)}=\frac{\text{Released Dox Amount (mg)}}{\text{Total Dox Amount (mg)}}\times 100\%\\ \text{Loading Efficiency(LE)}=\frac{\text{Released Dox Amount (mg)}}{\text{Particles Amount (mg)}}\times 100\% \end{array}$$

### Controlled release of DOX

DOX-loaded nanoparticles were re-suspended in PBS containing 0.02% sodium azide at 1 mg/ml in an Eppendorf tube and rotated in a low-speed shaker at room temperature. Release of DOX was monitored at several time intervals over 14 days. At each sampling time, the nanoparticle suspension was centrifuged for 10 min at 13,000 rpm. The supernatant was removed for quantification of DOX and an equal volume of PBS was replaced for continued monitoring of release. Detection of DOX was performed using the same methods as described above.

### Cytotoxicity evaluation

4T1 cells were plated in a 96-well cell culture plate at a concentration of 2×10^3^ per well and incubated with concentrations of nanoparticles ranging from 1.25 to 500 ug/mL. The same amounts of free DOX were added to parallel wells as controls. Three days after treatment, the effect of treatments on cell proliferation was determined using the standard MTT assay.

### DNA binding by 3E10^EN^-conjugated nanoparticles

Plasmid DNA pGL4.74 (20 ug, Promega) was linearized using BamHI to expose phosphate groups, which was next activated by N-(3-Dimethylaminopropyl)-N′-ethylcarbodiimide hydrochloride (EDC, 0.20 mg, 1.0 umol) and N-Hydroxysuccinimide (NHS, 0.17 mg, 1.5 umol) and reacted with NH2-PEG50-NH2 (0.41 mg, 3.0 umol). The resulting DNA with amine groups was then thiolated with Traut's Reagent (0.68 mg, 5.0 umol) and coated to a glass plate surface functionalized with maleimide groups (MicroSurfaes, Inc). To determine the binding ability of nanoparticles, IR780-loaded nanoparticles with and without 3E10^EN^ were re-suspended in PBS at 1 mg/ml and added to the glass plate surface. After a 1 hour incubation at room temperature, the glass plate was rinsed with water. Nanoparticles attached on the glass plate were detected at 745 nm/ 800 nm (Ex/Em) using an in vivo imaging system (IVIS) system (Xenogen).

### Measurement of exDNA in tumors and healthy tissues

Tumors were excised from mice with or without treatment of 3E10^EN^/DOX-NPs. The livers, hearts and muscles harvested from healthy mice without tumors were used as controls. Tumors and control healthy tissues were sliced in a similar size, mounted to a slide, and stained with Picogreen (ThermoFisher Scientific). Five minutes later, the slide was rinsed with water. The fluorescence intensity was detected at 465 nm/ 520 nm (Ex/Em) by the IVIS.

### *In vivo* tumor homing of nanoparticles

Female BALB/c mice (Charles River Laboratories) were used for this study and maintained in a sterile environment. This project was approved by the Yale University Institutional Animal Care and Utilization Committee (IACUC). To establish tumors, mice received subcutaneous flank injections of 1×10^6^ 4T1 tumor cells. Tumor size was measured weekly using traceable digital vernier calipers (Fisher). Tumor volume was determined by measuring the length (l) and width (w), and then calculating the volume (V) using the following formula: *V* = *lw*^2^/2. Tumor homing study was started when the volume reaches ~200 mm^3^ (day1). Mice were randomly divided into 3 groups. The first group was treated with 3E10^EN^-conjugated nanoparticles without IR780 at day 1 and 2 and received a final injection of 3E10^EN^-conjugated, IR780-loaded nanoparticles at day 3. The second and third groups received intravenous administration of IR780-loaded nanoparticles and 3E10^EN^-conjugated, IR780-loaded nanoparticles at day 3. Nanoparticles were administered at 1 mg per mouse. The loadings of IR780 in IR780-loaded nanoparticles and 3E10^EN^-conjugated, IR780-loaded nanoparticles were comparable. On day 5, mice were euthanized and the tumors were harvested for imaging using the IVIS. After imaging, tumors were lyophilized and homogenized in DMSO. The amount of dye in tumors was extracted and quantified using a microplate (BioTek).

### Antitumor evaluation in mouse tumor xenografts

Mice bearing 4T1 tumors were established as described above. In this tumor study treatments began when tumor volumes reached ~100 mm^3^. Mice were randomly divided into five groups, with seven mice per group, as follows: group 1 received treatment with PBS; group 2 received treatment with free DOX in PBS; group 3 received treatment with 3E10^EN^-NPs; group 4 received treatment with DOX-NPs; and group 5 received treatment with 3E10^EN^/DOX-NPs. Treatment was performed 3 times per week. Nanoparticles were administered at 1 mg, equal to 60 ug DOX per mouse. Tumor sizes were measured three times a week. Mice were euthanized when tumor volume reached ~1000 mm^3^, at which point the tumors were excised and fixed in formalin for immunohistochemistry. Serial sections were obtained and stained with hematoxylin and eosin (H&E) and Terminal Deoxynucleotidyl Transferase (TUNEL) for analysis of therapeutic effect. The growth curve was plotted using the mean of the tumor volumes for each treatment group, at each timepoint.

### Statistical analysis

Data were taken in triplicate and reported as mean with standard deviation. Comparison of the DNA binding ability of nanoparticles, the amount of exDNA in tissues, and the nanoparticle delivery efficiency between two conditions was evaluated by a paired Student's t-test. One-way ANOVA analysis was performed to determine the statistical significance of treatment related changes in tumor volume. A *p* ≤ 0.05 was considered to indicate a statistically significant difference.

## SUPPLEMENTARY FIGURES


