# Supplementary material for: A lupus anti-DNA autoantibody mediates autocatalytic, targeted delivery of nanoparticles to tumors
Source: Oncotarget. 2016 Aug 2;7(37):59965–75. doi: 10.18632/oncotarget.11015 (PMC5312362; doi:10.18632/oncotarget.11015)
Supplement: Supplementary file 1 [file oncotarget-07-59965-s001.pdf]

A lupus anti-DNA autoantibody mediates autocatalytic, targeted delivery of nanoparticles to tumors

SUPPLEMENTARY FIGURES

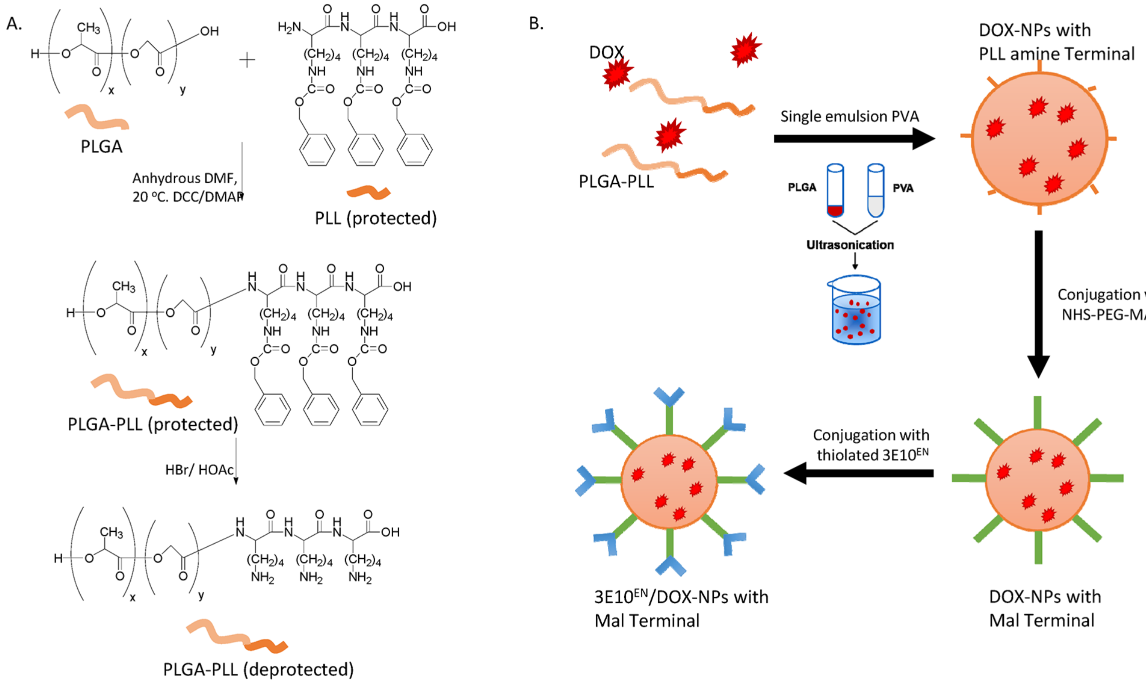

Supplementary Figure S1: Schematic diagrams of PLGA-PLL synthesis and 3E10^{EN}/DOX-NP fabrication. A. Reaction scheme for PLGA-PLL polymer. **B.** Procedures for 3E10^{EN}/DOX-NPs synthesis. Detailed methods are described in Materials and Methods.

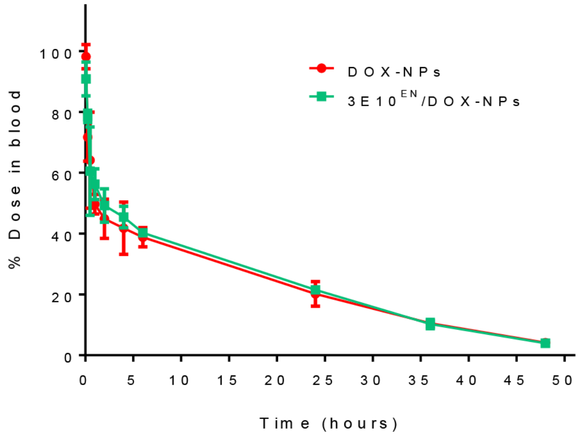

Supplementary Figure S2: Blood concentrations of DOX-NPs with and without conjugation of 3E10^{EN} recorded as a function of time. To enable detection of NPs, NPs were co-loaded with 1% coumarin 6. Six BALB/c mice were randomly assigned to two groups, which received intravenous administration of 1 mg DOX-NPs with and without conjugation of 3E10^{EN}, respectively. At 5 min, 15 min, 30 min, 1 h, 2 h, 4 h, 6 h, 24 h, 36 h and 48 h post injection, 20 uL blood sample from each mouse was collected into a 1.5 mL Eppendorf tube and lyophilized. Then, 100uL DMSO and 1 mL acetonitrile were added to each tube. After sonication in a water bath sonicator for 15 min, samples were subjected to centrifugation at 4000 rpm for 20 min to remove cellular fragments and blood albumin. Then, 0.8 ml supernatant from each sample was collected and added to an eppendorf tube. The acetonitrile was evaporated and the dye in the DMSO was quantified at Ex/Em 444/505 nm using a plate reader (BioTek ELx800). Data are shown as mean ± SD (n = 3).
